# Increased *RPA1* Gene Dosage Affects Genomic Stability Potentially Contributing to 17p13.3 Duplication Syndrome

**DOI:** 10.1371/journal.pgen.1002247

**Published:** 2011-08-25

**Authors:** Emily Outwin, Gillian Carpenter, Weimin Bi, Marjorie A. Withers, James R. Lupski, Mark O'Driscoll

**Affiliations:** 1 Human DNA Damage Response Disorders Group, Genome Damage and Stability Centre, University of Sussex, Brighton, United Kingdom; 2 Department of Molecular and Human Genetics, Baylor College of Medicine, Houston, Texas, United States of America; 3 Medical Genetics Laboratories, Baylor College of Medicine, Houston, Texas, United States of America; 4 Texas Children's Hospital, Houston, Texas, United States of America; 5 Department of Pediatrics, Baylor College of Medicine, Houston, Texas, United States of America; University of Pennsylvania, United States of America

## Abstract

A novel microduplication syndrome involving various-sized contiguous duplications in 17p13.3 has recently been described, suggesting that increased copy number of genes in 17p13.3, particularly *PAFAH1B1*, is associated with clinical features including facial dysmorphism, developmental delay, and autism spectrum disorder. We have previously shown that patient-derived cell lines from individuals with haploinsufficiency of *RPA1*, a gene within 17p13.3, exhibit an impaired ATR-dependent DNA damage response (DDR). Here, we show that cell lines from patients with duplications specifically incorporating *RPA1* exhibit a different although characteristic spectrum of DDR defects including abnormal S phase distribution, attenuated DNA double strand break (DSB)-induced RAD51 chromatin retention, elevated genomic instability, and increased sensitivity to DNA damaging agents. Using controlled conditional over-expression of *RPA1* in a human model cell system, we also see attenuated DSB-induced RAD51 chromatin retention. Furthermore, we find that transient over-expression of RPA1 can impact on homologous recombination (HR) pathways following DSB formation, favouring engagement in aberrant forms of recombination and repair. Our data identifies unanticipated defects in the DDR associated with duplications in 17p13.3 in humans involving modest RPA1 over-expression.

## Author Summary

The widespread use of genomic array technology has lead to the identification of a plethora of novel human genomic disorders. These complex conditions occur as a consequence of structural genomic alterations (deletions, amplifications, complex rearrangements). Understanding the specific consequences of such alterations on gene expression and unanticipated impacts on biochemical pathways represents an important challenge to help untangle the clinical basis of these conditions and ultimately aid in their management. Here, we demonstrate that individuals with specific duplications of 17p13.3 incorporating *RPA1* exhibit modest over-expression of RPA1. Unexpectedly, this is associated with elevated levels of genomic instability and sensitivity to DNA damage. RPA1 is a component of the Replication Protein A heterotrimer, a complex that plays fundamental roles in DNA replication, repair, and recombination. Reduced *RPA1* levels are associated with impaired DNA damage checkpoint activation, but the cellular impacts of over-expression of this subunit have not previously been described in the context of a genomic disorder. Using model cell and reporter systems, we show that modestly elevated levels of RPA1 can adversely impact on DNA double-strand break–induced homologous recombination resulting in elevated levels of chromosome fusions. This data highlights an unanticipated consequence of copy number variation on genomic stability.

## Introduction

Variously sized contiguous deletions within 17p13.3-pter are associated with complex clinical features in humans including structural brain abnormalities (lissencephaly, agyria, microcephaly), growth retardation and developmental delay [1]. Multiple pathogenomic studies have identified haploinsufficiency of genes including *PAFAH1B1* (LIS1) and *YWHAE* (14-3-3ε) as being particularly relevant in this context [2]–[5]. Previously, we have shown that patients with haploinsufficiency of *RPA1* exhibit defective ATR-dependent DDR including failure of the G2-M cell cycle checkpoint suggesting *RPA1* is sensitive to copy number variation [6]. Defective ATR-dependent G2-M arrest is associated with human conditions characterised by severe microcephaly (e.g. Seckel syndrome, Microcephalic primordial dwarfism type II, MCPH1-dependent Primary microcephaly, Nijmegen breakage syndrome) [7]. *RPA1* (RPA1: RPA-70KD) encodes the largest subunit of the Replication Protein A complex, a heterotrimeric complex (RPA1-2-3: RPA-70KD-RPA-32KD-RPA14KD respectively) with single stranded DNA binding capability that appears to be involved in multiple DNA transactions. It functions to prevent unregulated nuclease digestion and/or hairpin formation as well as orchestrating the sequential assembly and disassembly of various DNA processing factors during DNA replication, repair and recombination [8]–[10]. With respect to the DDR, the DNA single stranded binding function of RPA1–3 plays a fundamental role in the recruitment of ATR to sites of DNA damage, for example stalled replication forks, via a direct interaction with ATR's binding partner, ATRIP [11]. Furthermore, through interactions with RAD51 and RAD52, RPA1–3 also plays an essential role in homology directed recombinational repair, likely facilitating RAD51 nucleofilament formation allowing strand invasion and homology searching [12]–[16].

Recently, distinct, variously sized, non-recurrent duplications within 17p13.3 have been identified in several individuals defining a novel genomic disorder. In two of these the duplication included *RPA1* [17]. Consistent with other genomic disorders, the clinical duplication phenotype appears to be less severe compared to deletions within 17p13.3. Nevertheless, subtle over-expression of ‘normal’ genes within 17p13.3 is associated with profound clinical consequences [17]–[19]. Interestingly, over-expression of RPA1 has been implicated in genomic instability in other systems. For example, a quantitative over-expression screen in the budding yeast *Saccharomyces cerevisiae* found that over-expression of *RFA1*, the *S. cerevisiae* equivalent of mammalian *RPA1*, was associated with delayed cell cycle progression through G2-M, impaired chromosomal spindle attachment and activation of the DDR [20]. Furthermore, ectopic over-expression of individual RPA1–3 subunits in the human colorectal carcinoma cell line HCT116 promoted endoreduplication and aneuploidy [21]. Whether RPA1–3 over-expression functionally contributes to any cellular or clinical phenotype associated with genomic disorders has not been investigated. Since we had previously observed specific DDR-defects associated with reduced RPA1 expression in cell lines derived from individuals with variously sized contiguous deletions at 17p13.3-pter, we sought to determine if increased levels of RPA1 are associated with identical and/or related DDR-defects [6]. Herein, we show that cell lines derived from patients with 17p13.3 duplications that encompass *RPA1* exhibit modest RPA1 over-expression, abnormal S phase distribution, attenuated DSB-induced RAD51 chromatin retention and enhanced sensitivity to killing by camptothecin, consistent with compromised homologous recombination (HR). Using various model and reporter systems we demonstrate that subtle over-expression of RPA1 is indeed associated with altered HR-mediated DNA double strand break repair.

## Results

### Genomic duplications in 17p13.3 incorporating RPA1 are associated with RPA1 over-expression

**Figure 1:**
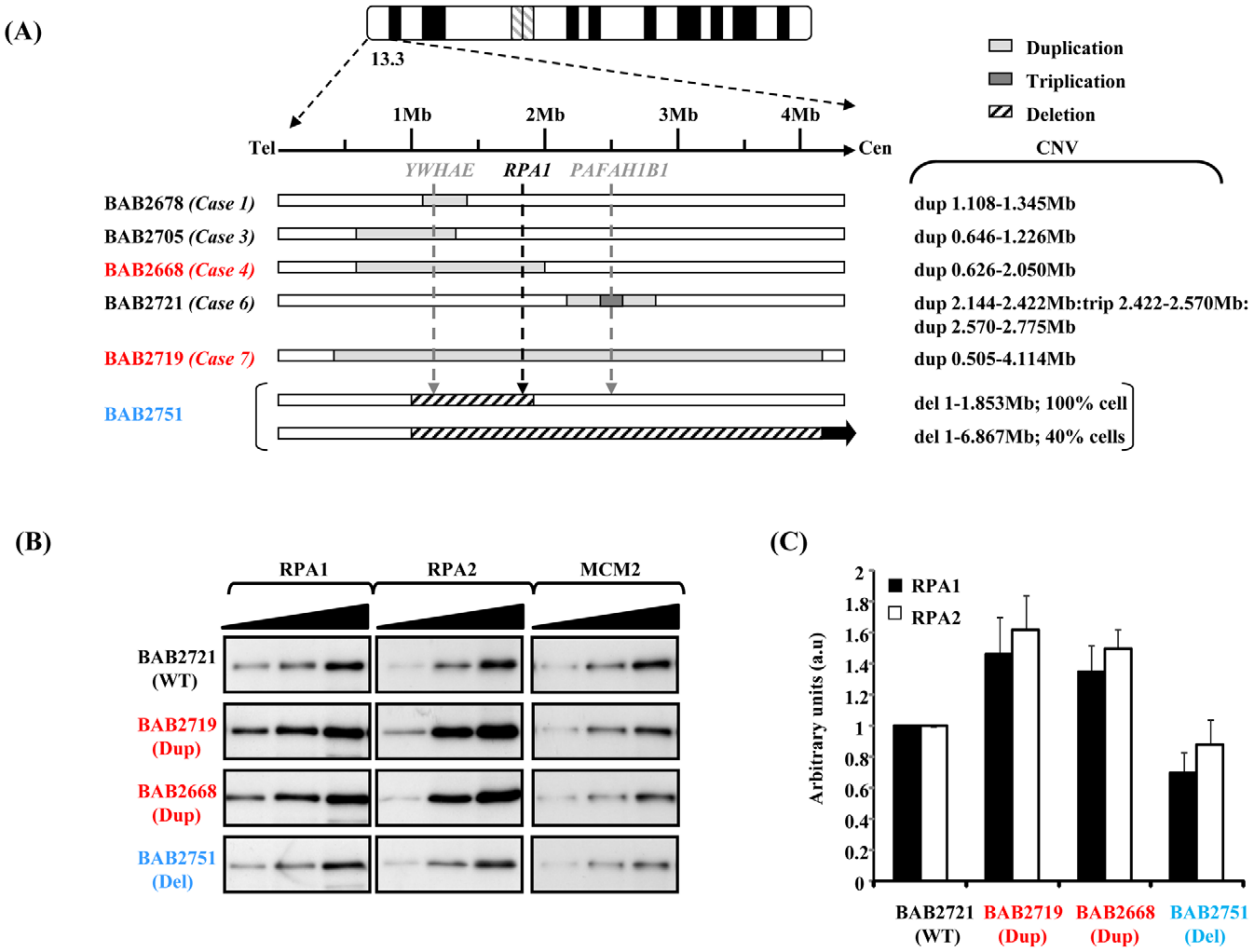
Duplication of *RPA1* results in RPA1 and RPA2 over-expression. (A) Schematic showing the copy number variation (CNV) of the various patient-derived lymphoblastoid cell lines (LBLs) used in this study. The *‘Case’* numbers serve as identifiers for the patients described by Bi *et al* [17]. The two lines associated with *RPA1* duplication are highlighted in red. BAB2751 (in blue) exhibits haploinsufficiency involving *RPA1* (in 100% of cells), as previously described by Nagamani *et al* [37]. This case also exhibits a heterozygous deletion of 1–6.867 Mb in 40% of cells by FISH [37]. (B) Western blot analysis for expression of RPA1 (left-hand panel) and RPA2 (middle panel) with MCM2 (right-hand panel) as a loading control. Whole cell extracts were prepared using urea-buffer from patient derived LBLs. BAB2721 (WT; wild-type *RPA1* copy number) is an LBL from a patient with a duplication in 17p13.3 not involving *RPA1* (case 6 Figure 1A). Both BAB2719 (Dup; *RPA1* duplication) and BAB2668 (Dup) exhibit duplications involving *RPA1*, whilst BAB2751 (Del; *RPA1* heterozygous deletion) exhibits *RPA1* haploinsufficiency. Each panel shows sequential loading of 2.5 µg, 5 µg and 10 µg extract. (C) Quantitative expression analysis of RPA1 and RPA2, standardised to MCM2 expression and normalised to BAB2721 (WT). Measurements were taken directly from the membrane during ECL development using the Image Quant LAS 4000 luminescent image analyser and Image Quant TL7.01 quantification software, so as to ensure signals were in the linear range.

Two of the 17p13.3 duplication cases recently described by Bi *et al* involve genomic duplication of *RPA1*, amongst other genes [17]. A schematic representation of the various CNVs in this region in several cell lines used in this study is shown in Figure 1A. The cell lines involving *RPA1* duplication, BAB2668 and BAB2719, are shown in red (Figure 1A).

We examined RPA1 expression by western blotting following careful titration of whole cell extracts to ascertain the extent of over-expression at the protein level using EBV-transformed lymphoblastoid cells (LBLs) from both *RPA1*-duplication cases, compared to another LBL with a 17p13.3 duplication that does not involve *RPA1* (BAB2721), as reported by Bi *et al* [17], and to an LBL (BAB2751; Case 6 [17]) exhibiting a novel 17p13.3 genomic deletion involving haploinsufficiency of *RPA1* (Figure 1B). The left-hand panel of Figure 1B shows that RPA1 protein is modestly over-expressed in whole cells extracts from BAB2668 (Case 4 [17]) and BAB2719 (Case7 [17]) compared to BAB2721, an LBL from a patient who does not exhibit *RPA1* duplication at the genomic level. LBLs from patient BAB2751 (Del; deleted for one copy of *RPA1*) associated with genomic haploinsufficiency of *RPA1* show modestly reduced RPA1 expression at the protein level. Interestingly, modest over-expression of RPA2 was also evident in whole cell extracts from BAB2719 (Dup) and BAB2668 (Dup) (Figure 1B middle panels). This suggests that the 17p13.3 duplications involving *RPA1* and resulting in RPA1 over-expression in these cells likely also results in elevated levels of the RPA complex since RPA2 levels appear modestly elevated in these cells (Figure 1B and 1C). Quantification of three separate experiments relative to MCM2 is shown in Figure 1C. Similar data from the other LBLs as described in Figure 1A is shown in Figure S1.

### Normal G2-M and spindle assembly checkpoint activation in 17p13.3 duplication syndrome associated with RPA1 over-expression

**Figure 2:**
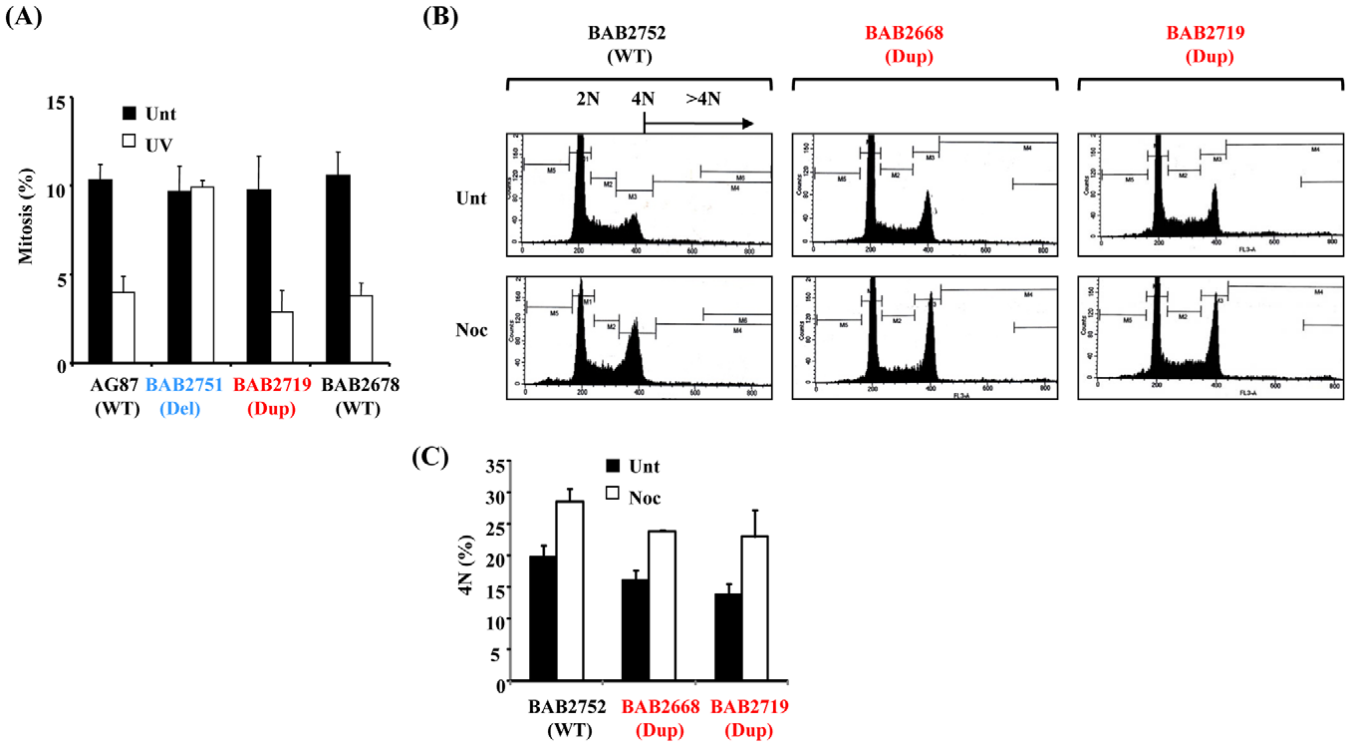
The ATR-dependent G2-M cell cycle checkpoint and the spindle assembly checkpoint (SAC) are functional in 17p13.3 duplication syndrome LBLs associated with *RPA1* duplication. (A) ATR-dependent UV-induced G2-M checkpoint arrest as determined by mitotic index (MI) is normal in LBLs associated with RPA1 over-expression, BAB2719 (Dup; *RPA1* duplication), or with normal wild-type (WT) *RPA1* copy number (AG87 and BAB2678), in contrast to those exhibiting RPA1 haploinsufficiency, BAB2751 (Del; *RPA1* heterozygous deletion). AG87 is a LBL from a normal wild-type (WT) individual. A functional checkpoint arrest is indicated in a reduced MI following UV-treatment. Unt; untreated, UV; UV-irradiated 5 J/m^2^ 24 hrs. Data represents the mean ± sd of three experiments. (B) Propidium iodide-derived flow cytometry profiles from LBLs either untreated (Unt) or following 24 hrs 1.5 µM nocodazole (Noc). BAB2752 (WT; wild-type) is an LBL from the unaffected parent of BAB2751 (Figure 1A), whilst BAB2668 (Dup; *RPA1* duplication) and BAB2719 (Dup; *RPA1* duplication) both exhibit RPA1 over-expression (Figure 1B). A functional SAC is indicated by increased 4N DNA content following nocodazole treatment (Noc) compared to untreated (Unt), without an associated increase in cells with >4N. (C) Quantification of the 4N DNA content of the untreated (Unt) and nocodazole treatment (Noc; 24 hrs 1.5 µM) LBLs shown in (B) showing a nocodazole-induced increase in all of the LBLs examined indicative of a functional SAC irrespective of *RPA1* copy number. Data represents the mean ± sd of three experiments.

An important role of RPA1–3 in the ATR-dependent DDR is the recruitment of ATR-ATRIP to single stranded DNA (ssDNA) generated at the DNA damage site, thereby initiating ATR-dependent signalling [11], [22]. One aim of this process is the activation of cell cycle checkpoint arrest, particularly at the G2-M transition. Previously, we have shown that Miller-Dieker Syndrome (MDS) and severe Isolated Lissencephaly Sequence (ILS+) patient-derived LBLs with *RPA1* haploinsufficiency fail to activate the ATR-dependent G2-M checkpoint [6]. Furthermore, we showed that this cellular phenotype was RPA1-dependent since it could be complemented by ectopic expression of *RPA1* following transfection [6]. Interestingly, precedent exists whereby over-expression of a DDR-component is actually associated with a functional defect in the DDR [23]–[28]. Nevertheless, we did not observe a defective ATR-dependent G2-M cell cycle checkpoint arrest in LBLs derived from individuals with increased RPA1 levels associated with *RPA1* duplication (Figure 2A). This was in contrast to LBLs exhibiting *RPA1* haploinsufficiency with reduced RPA1 expression (BAB2751; Del; deleted for one copy of *RPA1*. Figure 2A and [6]). Therefore, modest over-expression of RPA1 in the context of 17p13.3 duplication is not associated with the same ATR-dependent DDR-defect as that of *RPA1* haploinsufficiency.

Since ectopic over-expression of *RPA1* has previously been shown to induce other forms of genomic instability including aneuploidy, we examined spindle assembly checkpoint (SAC) proficiency following prolonged exposure to the spindle poison nocodazole in *RPA1*-duplicated patient-derived LBLs [21]. Following 24 hrs treatment with 1.5 µM nocodazole, cells with a functional SAC exhibit an increased 4N population without any progression to >4N, as demonstrated by the propidium iodide staining flow cytometry profiles shown in Figure 2B (Unt; untreated. Noc; nocodazole treated). Quantification of the 4N population, with or without 24 hrs treatment with nocodazole, demonstrates that BAB2752 (WT; wild-type *RPA1* copy number), BAB2668 (Dup; *RPA1* duplication) and BAB2719 (Dup) all exhibit a similar arrest at 4N following nocodazole (Figure 2C). No increase in >4N was seen in either of the *RPA1*-duplicaiton containing LBLs, BAB2668 or BAB2719 (Figure 2B). Hence, we observed a normal nocodazole-induced arrest at mitosis with 4N DNA content suggestive of a proficient SAC in this context.

### Abnormal S-phase distribution associated with RPA1 over-expression in 17p13.3 duplication syndrome

**Figure 3:**
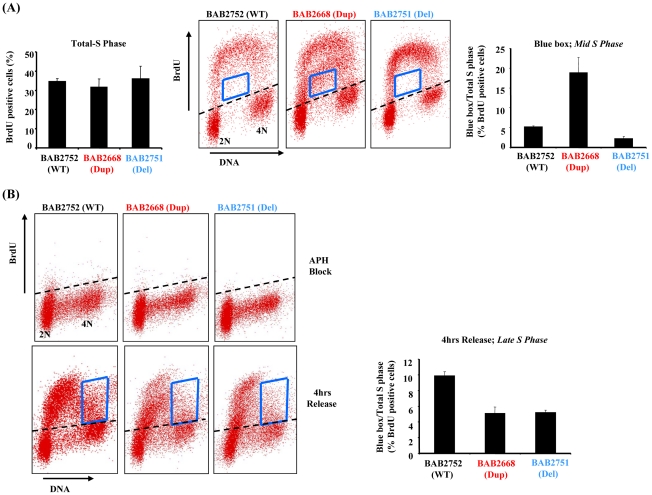
*RPA1*-duplication associated patient LBLs exhibit impaired DNA replication. (A) Left hand graph; Quantification of two dimensional flow cytometry using BrdU pulse labelling (15 mins in 50 µM BrdU) followed by immunodetection shows that the level of BrdU incorporation is equivalent for all LBLs tested irrespective of their *RPA1* copy number. Approximately 35–40% of cells from all lines incorporated BrdU following this pulse. Middle flow cytometry panels: The blue box indicates cells in mid-S phase (between 2N and 4N DNA content) that have inefficiently incorporated BrdU. Right hand graph: The relative amount of BrdU positive cells within the boxed area compared to total S phase (total BrdU positive cells). As a % of cells in S phase for each LBL following this pulse this amounts to approximately 5.7% for BAB2752 (WT), 18.7% for BAB2668 (*RPA1* duplication) and 2.7% for BAB2751 (*RPA1* haploinsufficient). Data represents the mean ± sd of three experiments. (B) Upper flow cytometry panels: Aphidicolin (APH; 10 µM 24 hrs) treatment dramatically inhibits BrdU incorporation equivalently in all LBLs as expected. Lower flow cytometry panels: 4 hrs after washing out of APH cells were pulse labelled with BrdU (15 mins 50 µM). The blue box indicates cells in late-S phase. Right hand graph: The relative amount of BrdU positive cells within the boxed area compared to total S phase (total BrdU positive cells). Both BAB2668 (*RPA1* duplication) and BAB2751 (*RPA1* haploinsufficient) LBLs show less incorporation of BrdU compared to BAB2752 (WT). Data represents the mean ± sd of three experiments.

Ectopic over-expression of *RPA1* is associated with endoreduplication in certain cell lines [21]. Since RPA1–3 complex is a fundamental component of normal DNA replication we examined S phase in one of our patient-derived LBLs with *RPA1* duplication (BAB2668) using bromodeoxyuridine (BrdU) pulse-labelling-coupled two-dimensional flow cytometry (Figure 3A). No evidence for spontaneous endoreduplication was found (*data not shown*). Whilst we did not observe a difference in the overall amount of BrdU incorporated between patient-derived LBL BAB2668 (Dup; with *RPA1* duplication) compared to those with normal (BAB2752;WT) or haploinsufficient *RPA1* copy number (BAB2751;Del) (Figure 3A left hand graph), the distribution or pattern of BrdU labelling was specifically and reproducibly altered in BAB2668 LBLs with *RPA1* duplication (Figure 3A middle flow cytometry panels). The BrdU positive cells within the boxed area represent those that have DNA content between 2N and 4N (mid S phase) but have not incorporated BrdU efficiently. These cells (i.e. mid S phase yet low BrdU incorporation) are approximately 3–4 fold more abundant in BAB2668 (Dup; *RPA1* duplicated) compared to BAB2752 (WT; *RPA1* copy no) or BAB2751 (Del; haploinsufficient *RPA1*) (Figure 3A right hand graph). This is suggestive of a stochastic problem in S phase progression or DNA replication in unperturbed asynchronously growing LBLs with *RPA1* duplication.

### RPA1 over-expressing 17p13.3 duplication syndrome cells exhibit delayed recovery from arrest in S-phase

We next examined the ability of our patient-derived LBLs to recover DNA replication following prolonged treatment with the DNA polymerase inhibitor aphidicolin (APH). APH treatment for 24 hrs efficiently reduced total BrdU incorporation as expected for all cell lines (Figure 3B upper panels). When this APH-induced DNA replication block was removed we found that RPA1-duplication associated LBL BAB2668 (Dup) failed to progress as efficiently as the control LBL BAB2752 (WT) through S phase, as judged by the distribution of BrdU positive cells in late S phase as indicated by the boxed area in Figure 3B (lower panels and graph). This is consistent with a constitutional problem in the ability to efficiently complete DNA replication, in this case following recovery from replicative stress, in these patient-derived cells. Interestingly, *RPA1* haploinsufficiency (BAB2751) conferred a similar phenotype (Figure 3B).

### Ionising radiation (IR)–induced RPA1, RPA2, and RAD51 chromatin retention is attenuated in RPA1 over-expressing 17p13.3 duplication syndrome cells

**Figure 4:**
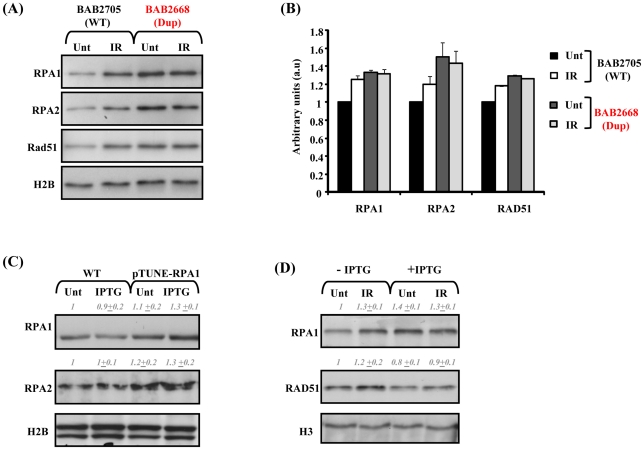
Modest RPA1 over-expression is associated with attenuated IR–induced RAD51 chromatin retention. (A) Impaired ionising radiation (IR)-induced RPA1, RPA2 and RAD51 chromatin retention in LBLs associated with *RPA1* duplication. LBLs with wild-type (WT) *RPA1* copy number (BAB2705) exhibit increased levels of chromatin bound RPA1, RPA2 and RAD51 following IR, in contrast to BAB2668 (Dup; *RPA1* duplication). Unt; untreated and IR; 24 hrs 10 Gy ionising radiation. Elevated chromatin bound RPA1, RPA2 and RAD51 is also evident in untreated (Unt) BAB2668 (Dup) relative to untreated (Unt) BAB2705 (WT), similar to the trend observed from whole cell extracts in Figure 1B and 1C. (B) Quantification of the relative expression of RPA1, RPA2 and RAD51 standardized to histone H2B levels and normalized to untreated (Unt) BAB2705 (WT; wild-type *RPA1* copy number). IR; ionizing radiation (10 Gy, 24 hrs). Data represents the mean ± sd of three experiments. Data was obtained using the Image Quant LAS 4000 luminescent image analyser. (C) T98G pTUNE-RPA1 containing cells (pTUNE-RPA1) exhibit a modestly increased basal level of RPA1 and RPA2 compared to the parental T98G line (WT) likely suggestive of significant inherent leakiness of this conditional system (Unt; untreated). Nevertheless, following isopropyl β-D-1-thiogalactopyranoside (IPTG) treatment (IPTG; 500 µM 3 hrs) a further subtle increase in RPA1 expression is detectable. Whole cell extracts were prepared by detergent lysis and blot signals were quantified on Image J, standardized to histone H2B loading and normalized to untreated (Unt) wild-type (WT). Data represents the mean ± sd from three separate blots. (D) pTUNE-RPA1 cells either without IPTG induction (−IPTG) or induced with IPTG (+IPTG) as in (C), were either left un-irradiated (Unt) or irradiated with 10 Gy ionising radiation (IR) and chromatin extracts prepared 24 hrs post-irradiation. Increased chromatin bound RPA1 and RAD51 was observed in the IR-treated (IR) non-induced (−IPTG) cells. IPTG induction (+IPTG) resulted in elevated RPA1 chromatin retention even in un-irradiated cells (Unt). These levels were not further elevated following IR-treatment (+IPTG, IR). Similarly, RAD51 chromatin retention was not affected following IR-treatment of the IPTG-induced cells (+IPTG, IR) relative to their un-irradiated counterpart (+IPTG, Unt). Although in this case, unlike in the patient-derived LBLs (A), spontaneously elevated chromatin bound RAD51 was not evident over that of the un-induced cells (−IPTG). Blot signals were quantified on Image J, standardized to histone H3 loading and normalized to untreated non-induced sample (−IPTG, Unt). Data represents the mean ± sd from three separate blots.

Since the RPA1–3 complex is also an important functional component of HR, we sought to examine HR in our patient-derived LBLs. Furthermore, defective HR has previously been shown to result in impaired S phase progression, a phenotype suggested by our BrdU incorporation data (Figure 3) [29], [30]. Following IR treatment, we found modestly increased chromatin binding of RPA1, RPA2 and RAD51 in LBLs with normal *RPA1* copy number (BAB2705; WT), in contrast to chromatin extracts from *RPA1*-duplicated BAB2668 LBLs (Dup) (Figure 4A and 4B). In fact, BAB2668 (Dup) exhibited increased endogenous levels of chromatin bound RPA1, RPA2 and RAD51, even in undamaged cells, in contrast to the WT LBLs, but this level did not change following IR (Figure 4A and 4B). Protein quantifications standardised to histone H2B loading and normalised to the un-irradiated BAB2705 (WT) for each of RPA1, RPA2 and RAD51 from three separate experiments are shown in Figure 4B. The attenuated IR-induced RAD51 chromatin retention observed in BAB2668 (Dup) indicates a potential problem in the ability to induce RAD51-dependent HR in these cells following IR-induced DSB formation.

Since the patient-derived LBLs with increased *RPA1* copy number are also duplicated for other genes, it was important to examine whether indeed RPA1 is the protein conferring this phenotype or whether it is in fact the consequence of combined increased copy number of several genes. To demonstrate that this cellular phenotype was specifically associated with *RPA1* over-expression, we constructed a conditional, isopropyl β-D-1-thiogalactopyranoside (IPTG)-inducible-*RPA1* model system in the human glioblastoma line T98G based on the pTUNE vector (Origene). Interestingly, we found that transient ectopic over-expression of RPA1 from a high expression level CMV-promoter-containing pcDNA3.1 mammalian expression vector was consistently associated with overt toxicity (associated with detectable activated caspase 3; *data not shown*) in multiple commonly employed human tumour lines (e.g. HeLa, MG63, A549) suggesting that strong over-expression of RPA1 is not tolerated. We found significant leakiness associated with the T98G-RPA1 system (pTUNE-RPA1) as RPA1 and RPA2 levels appeared increased even in the absence of IPTG (Figure 4C). Nevertheless, IPTG treatment did induce a modest elevated expression of RPA1 in this context (Figure 4C Unt; untreated. IPTG; IPTG treated). Interestingly, this was also associated with elevated RPA2 expression (Figure 4C), similar to what was found in the patient-derived LBLs (Figure 1B and 1C).

We found that the modest IPTG-induced over-expression of RPA1 was associated with attenuated IR-induced chromatin retention of both RPA1 and RAD51 (Figure 4D). Attenuated IR-induced RAD51 chromatin retention is also a feature of the patient-derived LBLs (Figure 4A and 4B). Furthermore, and similar to LBLs exhibiting RPA1 duplication (BAB2668; Figure 4A), we observed more chromatin associated RPA1 even in undamaged cells following induction with IPTG (Figure 4D). Collectively these data suggest that modest over-expression of RPA1 is associated with attenuated RAD51 chromatin recruitment following IR treatment and that this cellular phenotype is observed in LBLs from patients with duplications in 17p13.3 involving *RPA1* (Figure 4A).

### RPA1 over-expression affects I-Sce I–induced HR

**Figure 5:**
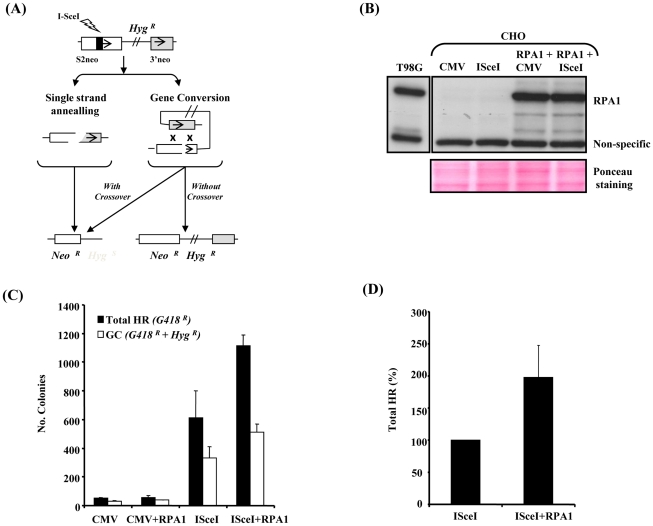
RPA1 over-expression results in a hyper-recombinogenic phenotype. (A) A schematic of the DRneo system for I-Sce I-induced HR based upon the reconstitution of a neomycin/G418 (Neo) cassette (S2neo joined to 3′neo). The retention of the hygromycin resistance gene (*Hyg^R^*) in this system distinguishes between gene conversion (GC) without crossing-over and alternative forms of recombination including single strand annealing (SSA) and GC involving cross-over (*intrachromatid crossing-over*). *Neo^R^*; G418 resistance. *Hyg^R/S^*; hygromycin resistance/sensitivity. The repair events monitored by DRneo include GC (*Hyg^R^+Neo^R^*), SSA (*Neo^R^*), GC involving intrachromatid crossing-over (*Neo^R^*) and non-homologous end joining although this will not involve recovery of an intact Neo gene and are not recovered here (*Hyg^R^*). Sister chromatid exchanges (SCE) are not usually an outcome of a two-sided DSB, hence SCEs are a very rare event in this reporter system. (B) RPA1 expression in the DRneo containing CHO cells following transfection with CMV-empty vector (CMV), I-Sce I-expression vector (I-Sce I) and/or human pcDNA3.1-RPA1. Detergent lysis extracts were used. The anti-RPA1 antibody used does not cross-react with hamster RPA1. The non-specific cross-reacting band qualitatively illustrates loading along with the Ponceau stained membrane shown in the lower panel. Endogenous RPA1 expression in T98G human glioblastoma cells is shown as a comparison to illustrate the extent of ectopic expression of human RPA1 in the various transfections. (C) I-Sce I-induced homologous recombination. CMV; empty vector, RPA1; RPA1 over-expression, I-Sce I; over-expression of I-Sce I. Cells containing a single integrated copy of the DRneo system were transfected as indicated and seeded onto plates in the presence of G418 or G418+hygromycin-B (Hyg). Colonies were scored 7–10 days post transfection. Data represents the mean ± sd of four experiments. (D) Relative level of total HR (G418 resistant colonies; *G418^R^*) following I-Sce I expression with and without RPA1 over-expression. RPA1 over-expression results in a 1.5–2 fold increase in recombination. Data represents the mean ± sd of four experiments.

To gain direct, independent insight into the consequences of subtle RPA1 over-expression on DSB-induced HR, we exploited I-Sce I restriction enzyme-induced HR using the established model DRneo reporter system in Chinese Hamster Ovary (CHO) cells following transient over-expression of human native untagged RPA1. Unlike other commonly used model HR reporter systems (e.g. DR-GFP), we opted to use this set-up specifically because the DRneo system enables the collective assessment of alternative forms of recombination alongside gene conversion (GC), such as single strand annealing (SSA) (Figure 5A) [31]–[33]. This is a heterologous system, although similar approaches have been used successfully before to study HR-mediated DSB-repair ([30] and references therein). Unfortunately, since the available RPA1 antibodies fail to cross-react with hamster RPA1, it was difficult to determine the precise extent of RPA1 over-expression following transient transfection. Nevertheless, transient expression of the human protein under these conditions appeared only modestly greater than endogenous RPA1 expression in T98G cells (Figure 5B). Interestingly, RPA1 expression in this context did not appear to grossly affect GC, although the limitations of such as heterologous system should be kept in mind (Figure 5C; *white bars*). Nevertheless, a reduced expression-induced RPA-dependent phenotype has been shown to give a different outcome using a similar system arguing against a simple dominant-negative effect here [16]. However, unexpectedly, we observed an approximately 2-fold increase in total levels of HR (i.e. all forms of GC+SSA; *black bars*) following I-Sce I-induced DSB formation (Figure 5C and 5D). This implies that RPA1 over-expression following I-Sce I-induced DSB results in increased forms of recombination such as SSA and/or GC with crossing-over which can be regarded as an *aberrant* or less favourable forms of recombination since they are associated with loss of genetic material [29]. Interestingly, RPA has recently been shown to be required for SSA in Xenopus [34]. Furthermore, increased RAD51 expression is also associated with increased genomic instability using a similar HR reporter system suggesting that over-expression of functional components of repair pathways likely to be involved in repairing such DSB's can adversely affect repair [35]. These data are consistent with attenuated IR-induced RAD51 chromatin recruitment observed in the *RPA1*-duplication associated patient-derived LBLs (Figure 4A) and the T98G-RPA1 system (pTUNE-RPA1; Figure 4D). Collectively, these results suggest that modest increased expression of RPA1 can influence HR sub-pathway choice.

### RPA1 over-expression affects genomic stability

**Figure 6:**
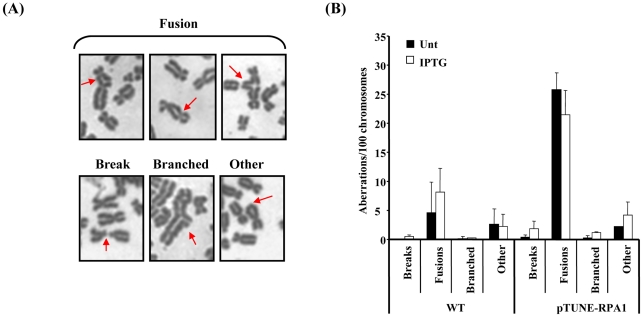
RPA1 over-expression is associated with elevated levels of chromosomal fusions. (A) Selected images denoting the various classes of chromosome aberrations observed using the pTUNE-RPA1 model system in T98G glioblastoma cells, including fusions between different chromosomes, breaks, branched structures and those categorised as ‘other’ typified by the class of terminal fusion shown here within a chromosome. (B) The pTUNE-RPA1 system exhibited elevated chromosomal instability even without treatment with IPTG (Unt; untreated) suggestive of significant leakiness in this system. Chromosomal fusions leading to derivatised chromosomes appeared to be the most frequent chromosome aberration observed in the pTUNE-RPA1 model system. Data represents the mean ± sd of three experiments. At least 1000 chromosomes were scored per sample.

Our findings with the model HR reporter system suggest that increased RPA expression was associated with *increased* recombination leading to the hypothesis that increased RPA expression could be associated with increased genome instability. Similarly, since RAD51 over-expression can also induce significant genomic instability [23]–[28], we examined mitotic spreads of our *RPA1*-over-expression model cell line (pTUNE-RPA1) for evidence of elevated genomic instability. Strikingly, significant levels of chromosome aberrations, fusions/derivatised chromosomes in particular, were observed in these cells (Figure 6A and 6B). Such abnormalities would be consistent with aberrant cross-over and/or ligation events. These aberrations were seen even without induction with IPTG which is a further indication of some inherent leakiness in this system and is consistent with elevated chromatin bound RPA1 seen here (Figure 4D). Despite the limitations of this artificial cell system, these data do demonstrate that subtle over-expression of RPA1 can induce significant levels of genomic instability, specifically elevated levels of derivatised chromosomes.

### Elevated genomic instability and DNA damage sensitivity in RPA1 over-expressing 17p13.3 duplication syndrome cells

**Figure 7:**
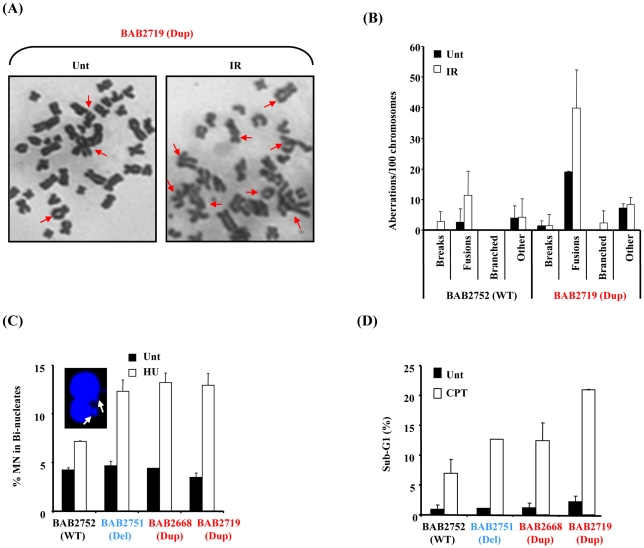
*RPA1*-duplication associated patient cells exhibit elevated genomic instability and sensitivity to DNA damage. (A) Chromosome spreads from untreated (Unt) or 24 hrs post 2 Gy ionising radiation (IR) from BAB2719 (*RPA1* duplication; Dup) showing multiple chromosome aberrations. Increased levels of complex chromosomal aberrations including fusions were consistently observed in BAB2719. (B) BAB2719 (*RPA1* duplication; Dup) exhibited increased levels of chromosome aberrations compared to BAB2752, an LBL with wild-type (WT) *RPA1* copy number, even in untreated (Unt) cells. These aberrations were further increased in the RPA1 over-expressing line (BAB2719) following IR-treatment as in (A). Similar to the pTUNE-RPA1 system, fusions and derivatised chromosomes appeared to be the most common chromosome aberration in BAB2719 associated with RPA1 over-expression (Dup; *RPA1* duplication) relative to the BAB2752 wild-type (WT; wild-type *RPA1* copy number). Data represents the mean ± sd of three experiments. At least 400 chromosomes were scored per sample. (C) HU-induced micronuclei (MN) were determined in bi-nucleate cells (image inset) for each LBL. Duplication of *RPA1* (BAB2668 and BAB2719) and/or haploinsufficiency of *RPA1* (BAB2751) was associated with increased levels of MN compared to BAB2752 wild-type (WT) *RPA1* copy number. Data represents the mean ± sd of three experiments. (E) Sensitivity to killing by CPT was determined by quantification of the sub-G1 population from propidium iodide flow cytometry profiles 72 hrs post treatment (CPT 10 µM). Duplication of *RPA1* (BAB2668 and BAB2719) and/or haploinsufficiency of *RPA1* (BAB2751) was associated with increased sensitivity to killing by CPT compared to BAB2752 wild-type (WT) *RPA1* copy number. Data represents the mean ± sd of three experiments.

To examine whether the patient-derived LBLs with RPA1 over-expression exhibited a similar phenotype we examined mitotic spreads for chromosomal abnormalities in LBLs from BAB2719 (Dup; *RPA1* duplication) compared to BAB2752 (WT; wild-type normal *RPA1* copy number). We also observed elevated levels of chromosomal aberrations, specifically an over-representation of chromosomal fusions, in LBLs associated with RPA1 over-expression (Figure 7A and 7B). These aberrations were increased following IR-treatment further suggestive of an inability of these cells to properly repair DSBs (Figure 7B).

Defective and/or aberrant DNA damage-induced HR is also associated with DNA damaging-induced genomic instability. Consistent with this we found increased levels of hydroxyurea (HU)-induced micronuclei (MN) formation in LBLs associated with *RPA1*-duplication (Dup; BAB2668, BAB2719) indicative of increased DNA breakage following replication fork stalling in these cells (Figure 7C). Furthermore, compromised HR is also specifically associated with sensitivity to killing by topoisomerase I inhibitors such as camptothecin (CPT) [36]. Consistent with an underlying problem with HR associated with *RPA1*-duplication in our patient-derived LBLs, we also found that these lines were sensitive to apoptosis induction following CPT treatment, as judged by increased levels of sub-G1 cells by propidium iodide flow cytometry (Figure 7D). Interestingly, for both of these cellular phenotypes we observed a similar response in BAB2751 (*RPA1* haploinsufficient) cells to that of lines over-expressing RPA1 (Dup; BAB2668 and BAB2719). This suggests that manipulation of RPA1 levels (increase or decrease) results in increased genomic instability following DNA damage.

In summary, we found that duplications involving *RPA1* are associated with modest over-expression of RPA1 and also RPA2 at the protein level, impaired S phase distribution and spontaneously elevated levels of chromatin bound RPA1, RPA2 and RAD51, along with attenuated IR-induced RAD51 chromatin retention following DSB's suggestive of compromised HR. Using the DRneo model HR-reporter system we observed a hyper-recombinogenic phenotype consistent with a shift towards a less genomically preferable form of HR following modest RPA1 over-expression. We also found increased levels of complex rearrangements especially after DSB-induction in patient derived LBLs with *RPA1* duplication. Furthermore, these patient derived cells exhibit other evidence of underlying problems in the DDR such as sensitivity to CPT and elevated HU-induced micronuclei formation.

## Discussion

Variously sized contiguous gene deletions at 17p13.3 are associated with severe neurodevelopmental phenotypes including microcephaly and neuronal migration deficits [1]. Recently, duplications within 17p13.3 have been identified in several patients exhibiting a milder though distinct phenotype that also incorporates aspects of autism spectrum disorder [17]–[19]. Much attention has focused on characterising the consequence of CNV of *PAFAH1B1/LIS1* in this respect [17]–[19], [37]. Previously, we have shown that LBLs from some ILS+ individuals and from MDS patients, all of whom exhibit haploinsufficiency of *RPA1*, a gene telomeric to *PAFAH1B1/LIS1*, exhibit impaired ATR-dependent DDR [6]. Here, we find that for the reciprocal situation, that is, in LBLs from patients associated with duplication of *RPA1*, we observed a distinct DDR abnormality impacting upon HR.

The RPA1–3 complex is a fundamental functional component of many DNA processes involving the generation of single stranded DNA [8]. RPA1–3 complex is essential for several DNA repair pathways (e.g nucleotide excision repair, mismatch repair, base excision repair), for DNA replication and recombination events [12]–[16]. Therefore, a plausible assumption would be that a significant reduction in RPA expression/function results in embryonic lethality. Attempts to create knockout mice for RPA1 have not been reported. Nevertheless, mice bearing a *semi-dominant* heterozygous mis-sense mutation in *Rpa1* (*Rpa1^L230P^*) exhibit gross genomic rearrangements and are highly cancer prone (*Rpa1^L230P^* homozygosity is cell lethal) [38]. Hence, precedent exists for altered RPA1, and likely, consequently RPA complex function, impacting on genomic stability at the organismal level. Furthermore, forced over-expression of RPA1 can cause genomic instability, at least in cancer cell lines [21].

There are several instances whereby over-expression of various DDR and/or cell cycle components disrupts or adversely affects the fundamental cellular processes/pathways in which they are functional components. For example, over-expression of CDC25A phosphatase is thought to be an important contributor to uncontrolled cell cycle progression from G2 into M frequently observed in certain malignancies [24], [25]. Over-expression of RAD51 and RAD52 has been found to reduce DSB-induced HR in mammalian cells [28]. Indeed, over-expression of separase or the SAC component MAD2 results in aneuploidy and malignancy in mice, consistent with defective SAC activity [26], [27]. Our findings suggest that a modest over-expression of RPA1 in LBLs derived from individuals with duplications in 17p13.3 involving *RPA1* results in an abnormal distribution of cells in S phase, adversely impacts on HR and is associated with elevated chromosomal instability and sensitivity to DNA damaging agents.

**Figure 8:**
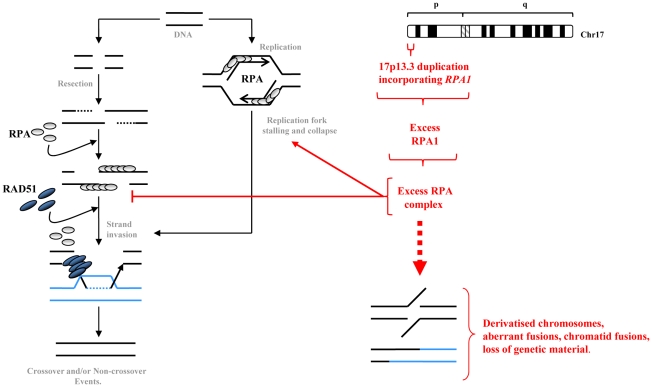
A summary model of how elevated RPA1 may adversely impact on homologous recombination. Homologous recombination (HR) is engaged when an overt DSB occurs at certain phases of the cell cycle or following replication fork stalling, say upon encountering a single strand break, or after overt replication fork collapse. RPA heterotrimeric complexes are displaced by RAD51 to facilitate strand invasion and homology searching. Our data indicates that modestly increased RPA1 levels as occurs in LBLs from individuals exhibiting duplication of *RPA1*, is also associated with elevated RPA2 suggestive of increased levels of RPA complex. This excess RPA complex, perhaps by sequestering binding partners, can result in delayed S phase progression and impact upon HR sub-pathway choice, potentially favouring more *aberrant* forms of recombination such as SSA and/or GC with crossing-over thereby promoting spurious fusions likely associated with loss of genetic material. This subtly elevated RPA1 level and consequently elevated RPA complex can result in elevated levels of chromosomal instability, derivatised chromosomes and enhanced sensitivity to DNA damaging agents.

RPA1 is thought to be of particular importance for RPA heterotrimeric function since it can bind DNA independently of the other subunits and contains the greatest surface area available to mediate protein-protein interaction [39]. Interestingly, we found that RPA2 also appeared to be over-expressed in our patient-derived LBLs associated with *RPA1* duplication, potentially suggesting elevated levels of RPA complex in this context. This could have adverse implications for coordinating subsequent DNA processing pathways. For example, during HR, a ‘handover’ between RPA1–3 complex coated ssDNA and RAD51 must occur to allow RAD51 nucleofilament formation for strand invasion. As RPA1 can bind RAD51 directly, an excess of chromatin bound RPA complex could interfere with the timing, coordination and/or efficiency of this ‘handover’ (Figure 8). A direct consequence of this could be either uncontrolled elevated or reduced overall HR capacity and/or a preference for other forms of recombinational repair, aside from gene conversion (GC). Data from our patient-derived LBLs show spontaneously elevated RAD51 on chromatin but attenuated IR-induced recruitment. Furthermore, our data generated following transient RPA1 over-expression in the DRneo system indicates a hyper-recombinogenic phenotype (i.e. total HR increases whilst levels of GC without crossing-over remain fairly constant). Interestingly, RPA has recently been shown to be required for SSA, at least in Xenopus [34]. One possible interpretation of the DRneo system-derived data is a shift towards an elevated level of SSA and/or GC with crossing-over, both of which involved loss of genetic material. The elevated levels of derivatised chromosomes observed in mitotic spreads from the *RPA1*-duplication associated LBLs and in the pTUNE-RPA1 system cells are consistent with aberrant cross-over and/or ligation events. A hyper-recombinogenic phenotype can have serious consequences for genome stability. For example, elevated ‘mutagenic’ HR has been implicated as pathophysiological contributor to disease progression in haematological malignancies such as Chronic Myelogenous Leukaemia and Multiple Myeloma [40]–[42].

The complex clinical spectrum of 17p13.3 microduplication syndrome is undoubtedly a consequence of the combined increased copy number of several genes within 17p13.3, although some candidates may have greater impacts than others [17]–[19]. The specific pathological connection between increased RPA1 expression and the clinical features of those respective patients is unclear. Nevertheless, the cellular phenotypes described here, including impaired S phase and suboptimal HR, could adversely influence apparently unrelated biological pathways by affecting gene expression. For example, components of the DNA replication machinery have been shown to influence epigenetic control of gene silencing [43]. Furthermore, suboptimal/aberrant HR could conceivably ultimately alter the genomic architecture resulting in unanticipated *cis* and/*trans* effects on the expression of other genes. Interestingly, *RPA1* has been implicated in such ‘*allelic phasing*’ together with *TP53* with respect to carcinogenesis [44]. Congenitally elevated genomic instability is often associated with cancer predisposition, although this has not been noted in either 17p13.3 duplication syndrome patients associated with *RPA1*-duplication [17]. Obviously there are too few patients to make any definitive conclusions, although the cellular defects presented here may warrant consideration in this respect. Clearly, further work is required to untangle the clinical consequences of increased RPA1 expression.

In conclusion, we have found that LBLs derived from patients with duplications in 17p13.3 specifically incorporating *RPA1* exhibit a modest over-expression of RPA1 and RPA2 which is associated with attenuated S phase transit, attenuated IR-induced RAD51 chromatin recruitment, elevated chromosomal instability, increased HU-induced MN formation and sensitivity to killing by CPT. All of these phenotypes are consistent with an inefficient HR pathway. Furthermore, using various model cell systems we showed that modest conditional over-expression of *RPA1* alone impacts on IR-induced RAD51 chromatin retention and I-SceI-induced HR in a reporter construct, the latter phenotype indicative of a hyper-recombinogenic shift towards alternative forms of recombination coincident with elevated chromosomal fusions. Collectively, our findings highlight a novel association between impaired DDR and CNV resulting in copy number gain of *RPA1*.

## Materials and Methods

### Cell lines

EBV-transformed patient-derived lymphoblastoid cell lines (LBLs) were cultured in RPMI with 15% FCS, L-Gln and antibiotics (Pen-Strep) at 5% CO_2_. T98G glioblastoma cells were maintained in MEM supplemented with 10% FCS, pyruvate and non-essential amino acids. Chinese Hamster Ovary cell lines (CHOs) were cultured in 10% DMEM, L-Gln and antibiotics (Pen-Strep) at 5% CO_2_.

### Whole cell extracts

Urea extraction: Cells were lysed in 150 µl urea buffer (9 M urea, 50 mM Tris-HCl at pH 7.5 and 10 mM 2-mercaptoethanol), followed by 15 s sonication, 30% amplitude using a micro-tip (SIGMA-Aldrich). The supernatant was quantified by Bradford Assay.

Detergent lysis: Cell pellets were incubated for 1 hr on ice in buffer containing 50 mM Tris-HCl pH 7.5, 150 mM NaCl, 2 mM EDTA, 2 mM EGTA, 25 mM NaF, 25 mM β-glycerolphosphate, 0.1 mM Na-orthovanadate, 0.2% Triton X-100, 0.3% IGEPAL and protease inhibitor cocktail tablets as indicated by manufacturer (Roche). The supernatant was quantified by Bradford Assay.

### Chromatin extracts

Cells were harvested 24 hr after 10 Gy gamma irradiation. Gamma irradiation was performed using a ^137^Cs γ-ray source at a dose rate of 8 Gy/min. Cells were lysed in detergent lysis buffer (above) for 1 hr on ice followed by 15 min in high-salt IP buffer (an extra 500 mM NaCl added to the regular IP buffer). The cell pellet was re-suspended in urea buffer (see WCE above) and sonicated for 15 s. The supernatant was quantified by Bradford Assay.

### Protein quantification from Western blotting

Western blots were developed using ECL (Pierce) in a luminescent image analyser, Image Quant LAS 4000 (GE Healthcare). This analyser ensures all bands are in the linear range (during the developing any saturated bands are highlighted so that the exposure can be decreased). Image Quant TL 7.01 quantification software was used to quantify the band intensities.

Alternatively, following ECL, Western blots were developed using film and the scanned images quantified with Image J software.

### Plasmids

Custom assembled pTUNE-RPA1 was obtained from Origene and stable T98G clones were obtained following transfection with MetafectenePro (Biontex Laboratories GmbH) and selection in G418 (1 mg/ml). For inductions, cells were treated with 500 µM IPTG for 3 hrs.

### Antibodies

Anti-RPA1 (Ab-1 #NA13) and anti-RPA2 (Ab-2 #NA18) antibodies were from Calbiochem. Anti-RAD51 (H-92) was from Santa Cruz. Anti-H3 was from Cell Signaling and anti-H2B was from Millipore. Anti-BrdU-FITC conjugated antibody (347583) was from Becton Dickinson.

### G2-M cell cycle checkpoint

UV irradiation was carried out using a UV-C source (0.6 J/m^2^/s). Cells were irradiated with 5 J/m^2^ UV-C in PBS and immediately seeded into complete medium supplemented with 1.5 µM nocodazole for 24 hr. Cells were pelleted, swollen with 75 mM KCl for 10 min before fixing in Carnoy's solution (methanol: glacial acetic acid 3∶1), before counterstaining with 4′-6-Diamidino-2-phenylindole (DAPI). Cells were Cytospun (Shandon) onto poly-L-lysine coated slides and mounted with Vectashield (Vector Labs). Slides were scored using a Zeiss AxioPlan microscope.

### Spindle assembly checkpoint

Exponentially growing LBLs were treated with 1.5 µM nocodazole for 24 hrs then fixed in 70% ice cold ethanol prior to propidium iodide staining and analysis by flow cytometry.

### Flow cytometry

LBLs were fixed in ice-cold 70% ethanol for 24 h and re-suspended in PBS containing 0.5% Tween-20, 10 µg/ml propidium iodide and 500 µg/ml RNase A. Data were collected using a Becton Dickinson FACS Calibur machine and were analysed with CellQuest software. For BrdU incorporation cells were labelled with 50 µM BrdU for 15 min. Incorporated BrdU was detected using FITC-conjugated anti-BrdU antibody (Becton-Dickson).

### DRneo system and homologous recombination

ERCC1.17 DRneo CHO cells were grown in 10% DMEM supplemented with 3 µg/ml Blasticidin-S and 0.05 mM hygromycin B [33]. Assay: 5×10^5^ cells were seeded in 6 cm plates. Next day cells were co-transfected with 2 µg RPA1 and 2 µg I-SceI (or CMV control) using MetafectenePro, according to the manufacturer's protocol. 24 hr after transfection cells were put into selection. For the recombination frequencies, 5×10^4^ cells per 10 cm plate were seeded with 1 mg/ml G418 and/or 0.5 mg/ml hygromycin-B. 10^3^ cells were seeded to determine the cloning efficiency. Plates were incubated for 7 days, after which they were stained with methylene blue.

### DNA damage sensitivity

Exponentially growing LBLs were treated with 10 µM camptothecin (CPT) and incubated for 72 hrs, fixed (ice cold 70% ethanol), stained with propidium iodide and sub-G1 cells quantified by flow cytometry.

### Micronuclei

Cells were treated with 1 mM HU for 4 hrs before incubation for 24 hrs in 5 µg/ml cytochalasin B for bi-nucelate formation. Micronuclei were scored in bi-nucleated cells by immunofluorescence microscopy (Zeiss AxioPlan) following swelling in KCl (75 mM 10 mins), fixation (Carnoy's fix; 3∶1 methanol∶acetic acid. 10 mins) and staining with DAPI and acridine orange (2 µg/ml).

### Chromosome analysis

The pTUNE-RPA1 T98G cells, and wild type T98G cells, were induced with 500 µM IPTG for 3 hr before adding 0.2 µg/ml colcemid for 4 hr prior to harvesting. Cells were swollen (75 mM KCl 10 mins) and then fixed in Carnoy's fixative (10 mins) prior to being dropped onto slides from approx 50 cm above. The slides were air dried and Giemsa stained according to the manufacturer's (Sigma) protocol. Images were captured on a Zeiss AxioPlan microscope. Chromosomes spreads were scored blinded according to the following criteria; fusions between different chromosomes, breaks, branched structures and ‘other’ (a terminal fusion within a single chromosome). The results were represented as aberrations per 100 chromosomes, rather than per metaphase due to the aneuploid nature of T98G. LBLs were treated with 2 Gy ionising radiation (IR) and allowed to recover for 24 hrs. The IR treated (IR) and untreated control (Unt) cells were treated with 0.2 µg/ml colcemid for 4 hr prior to harvesting. Cells were swollen (75 mM KCl 10 mins), fixed (Carnoy's), Giemsa stained and analysed as above.

## Supporting Information

Figure S1 Duplication of *RPA1* results in RPA1 and RPA2 over-expression. Western blot analysis for expression of RPA1 (left-hand panel), RPA2 (middle panel) and MCM2 (right-hand panel) using urea-derived whole cell extracts from patient derived LBLs. LBLs with wild-type *RPA1* copy number are shown in black, those with *RPA1* duplication in red and those with *RPA1* haploinsufficiency in blue. BAB2705 (WT; wild-type *RPA1* copy number) and BAB2678 (WT; wild-type *RPA1* copy number) are LBLs from patients with a duplication in 17p13.3 not involving *RPA1* (see Figure 1A). Both BAB2719 (Dup; *RPA1* duplication) and BAB2668 (Dup) exhibit duplications involving *RPA1*, whilst BAB2751 (Del; *RPA1* heterozygous deletion) exhibits *RPA1* haploinsufficiency. Each panel shows sequential loading of 2.5 µg, 5 µg and 10 µg extract.
(TIF)
