# Supplementary figures and images for: Increased RPA1 Gene Dosage Affects Genomic Stability Potentially Contributing to 17p13.3 Duplication Syndrome
Source: PLoS Genet. 2011 Aug 25;7(8):e1002247. doi: 10.1371/journal.pgen.1002247 (PMC3161930; doi:10.1371/journal.pgen.1002247)

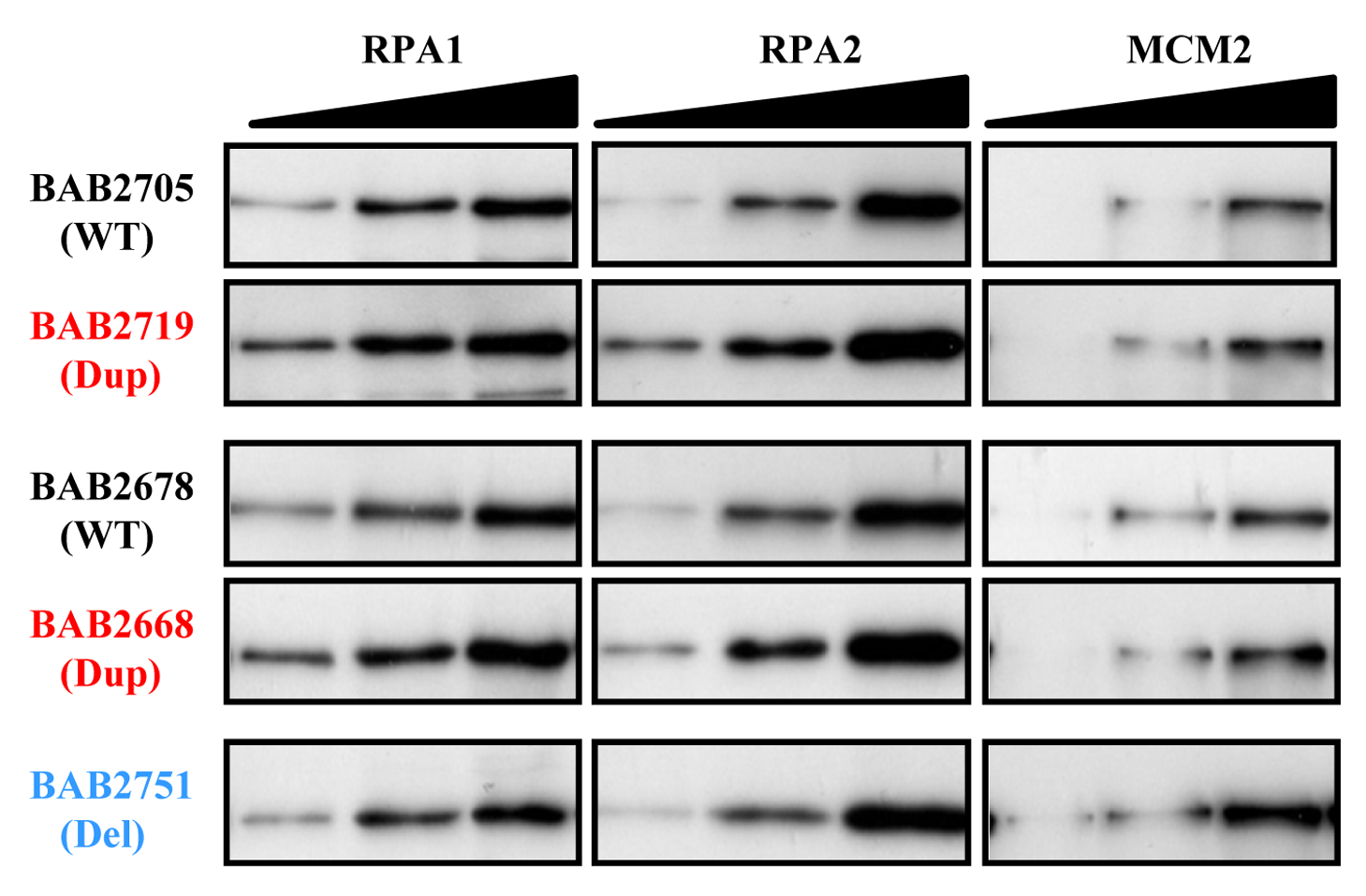

Supplement: Figure S1 — Duplication of RPA1 results in RPA1 and RPA2 over-expression. Western blot analysis for expression of RPA1 (left-hand panel), RPA2 (middle panel) and MCM2 (right-hand panel) using urea-derived whole cell extracts from patient derived LBLs. LBLs with wild-type RPA1 copy number are shown in black, those with RPA1 duplication in red and those with RPA1 haploinsufficiency in blue. BAB2705 (WT; wild-type RPA1 copy number) and BAB2678 (WT; wild-type RPA1 copy number) are LBLs from patients with a duplication in 17p13.3 not involving RPA1 (see Figure 1A). Both BAB2719 (Dup; RPA1 duplication) and BAB2668 (Dup) exhibit duplications involving RPA1, whilst BAB2751 (Del; RPA1 heterozygous deletion) exhibits RPA1 haploinsufficiency. Each panel shows sequential loading of 2.5 µg, 5 µg and 10 µg extract. (TIF) [file pgen.1002247.s001.tif]
